# Quantitative analysis of molecular partition towards lipid membranes using surface plasmon resonance

**DOI:** 10.1038/srep45647

**Published:** 2017-03-30

**Authors:** Tiago N. Figueira, João M. Freire, Catarina Cunha-Santos, Montserrat Heras, João Gonçalves, Anne Moscona, Matteo Porotto, Ana Salomé Veiga, Miguel A. R. B. Castanho

**Affiliations:** 1 Instituto de Medicina Molecular, Faculdade de Medicina, Universidade de Lisboa, Portugal; 2 Institut Pasteur, Unité de Virologie Structurale, Département de Virologie, F-75724 Paris Cedex 15, France; 3 Instituto de Investigação do Medicamento (iMed.ULisboa), Faculdade de Farmácia, Universidade de Lisboa, Portugal; 4 Laboratori d’Innovació en Processos i Productes de Síntesi Orgànica (LIPPSO), Departament de Química, Universitat de Girona, Spain; 5 Department of Pediatrics, Columbia University Medical Center, New York, USA

## Abstract

Understanding the interplay between molecules and lipid membranes is fundamental when studying cellular and biotechnological phenomena. Partition between aqueous media and lipid membranes is key to the mechanism of action of many biomolecules and drugs. Quantifying membrane partition, through adequate and robust parameters, is thus essential. Surface Plasmon Resonance (SPR) is a powerful technique for studying 1:1 stoichiometric interactions but has limited application to lipid membrane partition data. We have developed and applied a novel mathematical model for SPR data treatment that enables determination of kinetic and equilibrium partition constants. The method uses two complementary fitting models for association and dissociation sensorgram data. The SPR partition data obtained for the antibody fragment F63, the HIV fusion inhibitor enfuvirtide, and the endogenous drug kyotorphin towards POPC membranes were compared against data from independent techniques. The comprehensive kinetic and partition models were applied to the membrane interaction data of HRC4, a measles virus entry inhibitor peptide, revealing its increased affinity for, and retention in, cholesterol-rich membranes. Overall, our work extends the application of SPR beyond the realm of 1:1 stoichiometric ligand-receptor binding into a new and immense field of applications: the interaction of solutes such as biomolecules and drugs with lipids.

Biomolecular interactions with lipid membranes are relevant for several biological processes. Antimicrobial peptides^1^ and drug-carrying membrane-translocating vectors^2^, for instance, have led to an increasing interest in studying membrane-active molecules for their biological and pharmacological implications.

To study membrane association processes, it is essential to use adequate quantitative parameters, namely partition constants (*K*_p_), but also adapted methodology with optimized technical solutions. In recent years, the development of Surface Plasmon Resonance (SPR)-based techniques for biological applications has enabled real-time and label-free molecular binding detection^3^,^4^, including solute-lipid membrane interactions^5^. The widespread use of SPR methodologies is justified by the high sensitivity (sub-nanomolar), high-throughput capacity, and no need for labelling^6^. Applications to solute-membrane interaction studies with deposited lipid vesicles or lipid monolayers can be used to screen the membrane affinity of proteins, peptides or small molecules, for instance^7^,^8^,^9^,^10^. However, in contrast to the studies of 1:1 stoichiometric intermolecular receptor-ligand binding, there are no adequate mathematical models available for SPR lipid partition data treatment. In the absence of dedicated models, one-to-one (Langmuir) or two-state binding models are usually applied to the data but fail to quantitatively describe solute partition. Ultimately, binding constants (*K*_d_) are used instead of *K*_p_^11^.

Taking advantage of conventional SPR data, we have developed a quantitative SPR partition data analysis methodology based on two complementary mathematical models (steady-state and dissociation models). These were derived from canonical phase partition formalisms^12^,^13^ adapted to SPR membrane interaction experiments and integrated to allow broad range analysis of response data. Similar approaches have been accomplished for other methodologies, mainly fluorescence spectroscopy^13^, and correspond to a theoretical approximation of lipid membrane bilayers to a bulk lipid phase. The steady-state model allows *K*_p_ determination from sensorgram association phase response data when a maximum steady-state response is achieved. The dissociation model provides dissociation rate constants (*k*_off_) from kinetic evaluation of dissociation data. Both are applied to conventional sensorgram response data from commercial Biacore L1 sensor chip experiments.

The experimental design of the present method follows three distinct steps: First we evaluated the surface coverage of Biacore L1 sensor chips by lipid vesicles and vesicles integrity after deposition. This allows experimental validation of specific model assumptions. Then, in order to validate the *K*_p_ determination method, we applied the steady-state model to analyse the data of three distinct molecules of known *K*_p_: F63, an anti-human immune deficiency virus (HIV)-1 and 2 single-domain antibody^14^, enfuvirtide (ENF), an anti-HIV-1 fusion inhibitor peptide^15^, and kyotorphin (KTP), a small endogenous analgesic dipeptide^16^ (Supplementary Table S1). In addition to *K*_p_, the molar lipid:solute ratio at membrane saturation is retrieved. Lastly, the integration of steady-state and dissociation models was applied to the binding and unbinding of the HRC4 dimeric peptide, a novel antiviral fusion inhibitor against measles virus (MV) derived from a heptad repeat domain at the C-terminus of the MV fusion (F) protein^17^, designed to interact with lipid membranes (Supplementary Table S1). Not only were we able to determine the *K*_p_ towards three distinct lipid membrane compositions, we also determined HRC4 lipid-specific *k*_off_ rates and the fraction of peptide retained in the membranes. The influence of membrane-induced aggregation in the dissociation mechanism will be discussed.

## Methods

### Data analysis model development

A detailed description of the theoretical models and respective mathematical development is provided in the section 2 of the Supplementary information. Supplementary equations are numbered from (S1) to (S31). Equations in the main body are numbered independently.

### Chemicals and reagents

Recombinant F63 single domain antibody was obtained as described elsewhere^14^. ENF was a kind gift from Roche (Palo Alto, CA, USA). KTP was chemically synthesized as described elsewhere^18^. HRC4 was custom synthetized by American Peptide Company (Sunnyvale, CA, USA). 1-palmitoyl-2-oleyl-*sn*-glycero-3-phosphocholine (POPC), 1,2-dipalmitoyl-*sn*-glycero-3-phosphocholine (DPPC) and chicken egg sphingomyelin (SM) were purchased from Avanti Polar Lipids (Alabaster, AL, USA). Cholesterol (Chol) was from Sigma-Aldrich (St. Louis, MO, USA). 4-(2-hydroxyethyl)piperazine-1-ethanesulfonic acid (HEPES), NaCl, NaOH, DMSO and chloroform (the last two with spectroscopic grade) were obtained from Merck (Darmstadt, Germany). Fluorescent probes Rhodamine B conjugated with 1,2-dihexadecanoyl-*sn*-glycero-3-phosphoethanolamine (Rho-PE) and 5(6)-carboxyfluorescein (CF) were purchased from Thermo-Fisher Scientific (Waltham, MA, USA) and Sigma, respectively. Biacore sensor chip regeneration reagents 3-[(3-cholamidopropyl)dimethylammonio]-1-propanesulfonate (CHAPS) and methanol, were also from Sigma. Sodium dodecyl sulfate (SDS) was obtained from GE Healthcare (Little Chalfont, United Kingdom).

### Sample and Liposome Preparation

Lyophilized ENF and KTP were solubilized in 10 mM HEPES, 150 mM NaCl, pH 7.4 buffer to a 1 mg/mL stock solution concentration. Purified F63 stock solutions (~1 mg/mL) were prepared in the same buffer. Lyophilized HRC4 was first solubilized in DMSO to 40 mg/mL, then diluted in 10 mM HEPES, 150 mM NaCl, pH 7.4 buffer for the experiments. The final DMSO content was maintained at 2% (v/v) in all HRC4 experiments. ENF, KTP and HRC4 stock solutions were sonicated in an ultrasonic bath for a 10 min period.

Small unilamellar vesicles (SUV) suspensions were prepared as described before^19^,^20^. Briefly, the lipid mixture was dissolved in chloroform in a round bottom flask. Chloroform was evaporated under a constant nitrogen flow. The resulting lipid film was dried in vacuum, overnight. A multilamellar vesicle suspension (MLV) was obtained after lipid film rehydration with 10 mM HEPES, 150 mM NaCl, pH 7.4 buffer and a series of 10 freeze/thaw cycles. MLV suspensions were extruded through a 50 nm track-etched polycarbonate membrane from Whatman/GE Healthcare (Maidstone, United Kingdom). Extrusion was performed in a LiposoFast-Basic plus Stabilizer setup from Avestin (Mannheim, Germany). POPC, POPC:Chol (2:1), POPC:Chol:SM (1:1) and POPC:DPPC (1:1) mixtures were prepared. SUV hydrodynamic diameter (D_*H*_) and sample polydispersity were characterized through dynamic light scattering (DLS, Supplementary Fig. S1).

### Surface Plasmon Resonance

SPR experiments were carried out in a Biacore X100 apparatus from GE Healthcare. Temperature was set at 25 °C for all experiments. A 10 mM HEPES, 150 mM NaCl, pH 7.4 solution was used as running buffer for F63, ENF and KTP experiments. Alternatively, 10 mM HEPES, 150 mM NaCl, pH 7.4 containing 2% (v/v) DMSO was used for HRC4 experiments. The flow system was primed three times before initiating an experiment. The L1 sensor chip was used in all experiments. The sensor chip surface was rinsed with three injections of 20 mM CHAPS before SUV deposition. 1 mM lipid SUV samples were injected over the L1 sensor chip for 2400 s, at a 2 μL/min flow speed. Typical RU values for POPC, POPC:Chol (2:1), and POPC:Chol:SM (1:1:1) SUV depositions were ~8000, ~10000 and ~10500, respectively. Loose vesicles were removed with a 36 s injection of 10 mM NaOH at 50 μL/min. F63, ENF and KTP at defined concentrations (between 0.25 and 700 μM) were injected over pre-formed lipid vesicle-coated surfaces at 5 μL/min, for a total of 200 s (association phase). Samples were also injected at 30 μL/min to confirm the absence of mass transfer effects. Solutes were allowed to dissociate for 800 s. L1 sensor chip surface regeneration was performed with sequential injections of 20 mM CHAPS (5 μL/min for 60 s), 0.5% (w/v) SDS (5 μL/min for 60 s), 10 mM NaOH containing 20% (v/v) methanol (50 μL/min for 36 s) and 10 mM NaOH (50 μL/min for 36 s). Baseline response values were compared before and after each experiment to evaluate the effectiveness of the surface regeneration.

Raw SPR sensorgram data were collected for both lipid deposition and solute binding. SUV deposition response values were collected from sensorgrams upon reaching a stable response. For each studied molecule, association steady-state response values were collected from individual sensorgrams at *t* = 200 s. Dissociation response data were collected between 200 and 1000 s of each sensorgram.

### Confocal Fluorescence Microscopy and FRAP

POPC, POPC:Chol (2:1), POPC:Chol:SM (1:1:1), and POPC:DPPC (1:1) vesicles containing Rho-PE and CF were prepared. Rho-PE locates within the SUV membrane bilayer and CF in the lumen. The lipid mixture was co-solubilized with Rho-PE in chloroform to a final 1:125 probe to lipid ratio (0.8 mol% relative to lipids) to ensure adequate probe incorporation in both membrane leaflets. Rehydration was performed with a 25 mM CF solution in buffer to allow probe encapsulation within MLV. The subsequent SUV preparation steps were performed as previously described. Probe encapsulation was confirmed in each case (Supplementary Fig. S2). POPC SUV containing only the Rho-PE probe were used as a control.

Fluorescently labelled SUV were deposited in a L1 sensor chip following the previously described procedure. After deposition, the running buffer was allowed to flow for 30 min to remove non encapsulated CF. The sensor chip was undocked from the equipment and observed in a Zeiss LSM 710 confocal laser point-scanning inverted microscope from Carl Zeiss MicroImaging (Oberkochen, Germany) equipped with Diode 405–30, Argon, DPSS 561–10 and HeNe633 lasers. Images were taken directly on the L1 sensor chip surface using a Plan-Apochromat 10x and 20x objectives from Zeiss. Rho-PE and CF were excited with the DPSS 561–10 and Argon (488 nm) lasers, respectively. Z-stack images of SUV deposition on the L1 chip were collected to monitor the relative z-axis position of the membrane deposition and CF fluorescence emission within an 80 μm segment.

FRAP experiments were performed for each SUV lipid composition deposited on the L1 sensor chip using the following protocol^21^: 10 frames were collected before bleaching of a 20 μm radius circular region of interest (ROI). The 488, 514 and 561 lasers at 100% intensity irradiated the ROI for 100 iterations (approx. 40 s). After bleaching 90–150 images were acquired according to the lipid mixture studied and the fluorescence recovery/sample burning percentage, for a maximum of 850 s.

Experiments were performed at room temperature. All images were processed with the software ImageJ v1.49^22^.

## Results

### Validating L1 sensor chip coverage

In a standard SPR membrane interaction experiment, lipid SUV samples are injected on the hydrophobic L1 sensor chip surface composed of carbon chains, covalently bound to a dextran matrix. Through hydrophobic interactions, the vesicles gradually cover the dextran matrix and form a stable surface. Lipid surface coverage in L1 sensor chips has been previously studied, but there is no consensus on whether the captured lipid vesicles either remain intact anchored to the acyl chains, or fuse into planar stable lipid multilayers^23^,^24^.

**Figure 1:**
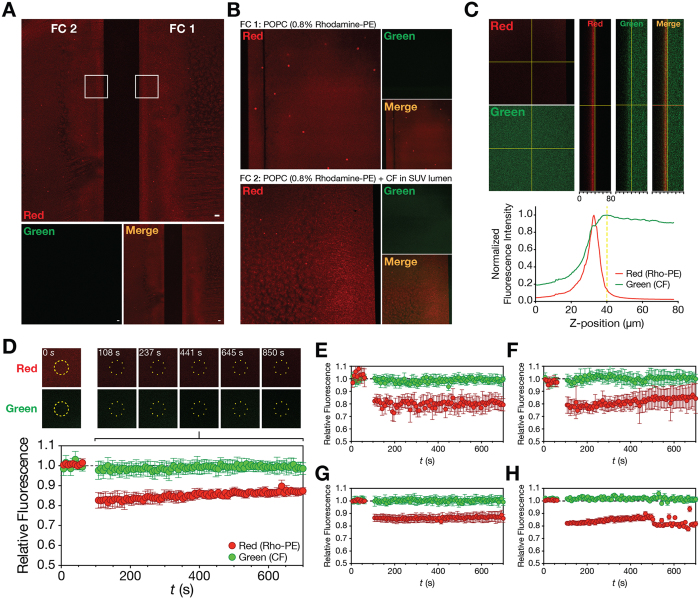
Characterization of the lipid surface formed on a L1 sensor chip. (**A**) Confocal images of flow cell (FC) 1 and 2 formed on the L1 sensor chip surface (XY plane) using 10x magnification objectives. POPC SUV labelled with either Rho-PE (control) or Rho-PE and CF were deposited on FC1 and 2, respectively. A 20 μm white scale bar is included. **(B)** Individual confocal images of FC1 (top) and 2 (bottom) collected at the respective white square ROI in **A** using a 20x magnification objective, emphasizing the flow cell boundaries. Red (Rho-PE), green (CF) and merge channels are depicted for both **A** and **B**. **(C)** Z-stack image of the L1 sensor chip covered with POPC SUV labelled with Rho-PE and CF. YZ orthogonal projections were produced in order to evaluate the relative Z-position of the lipid membrane and CF probe. A vertical yellow line is located in the middle of the Z projection (40 μm). Red (Rho-PE), green (CF) and merge channels are depicted. A normalized Z plot profile is also shown locating the region of the lipid deposition. **(D–H)** FRAP analysis of the circular ROI according to the settings explained in the Supplementary Fig. S3. Rho-PE and CF fluorescent signals were collected from the ROI and normalized to the signal on the non-bleached area. The relative fluorescence recovery (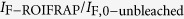) is thus shown to evaluate the lateral diffusion of the Rho-PE fluorescent lipids of the bilayer. Briefly, a time series of 4x Zoom 512 × 512 pixels images using a 20x magnification objective were taken: 10 frames were collected before bleaching of the 20 μm radius circular ROI (yellow) with 100 iterations (approx. 40 sec.) of 100% of 488, 514 and 561 laser intensity. After bleaching, 90–150 images were acquired according to the lipid mixture studied (**D** – POPC, **E** - POPC:Chol (2:1), **F** - POPC:Chol:SM (1:1:1), **G** - POPC:DPPC (1:1), **H** - POPC (control)) due to fluorescence recovery/sample burning percentage. Representative confocal images are presented in order of their acquisition time. Error bars correspond to the standard deviation of the mean. Experiments were performed in triplicate.

Due to the relevance of the sensor chip surface coverage and vesicle integrity to the application of the data analysis models, we have characterized different lipid surfaces formed on the Biacore L1 sensor chip using confocal fluorescence microscopy. Fluorescently labelled POPC, POPC:Chol (2:1), POPC:Chol:SM (1:1:1) and POPC:DPPC (1:1) vesicles, containing Rho-PE and CF, were used. These probes allowed monitoring of vesicle membranes and lumen, respectively. POPC:DPPC (1:1) vesicles were studied as a model of low fluidity lipid membranes. Additionally, POPC vesicles containing Rho-PE but not CF were used as a control. Through 2D confocal images, we were able to detect the fluorescence signal of Rho-PE in the lipid membranes deposited on the L1 sensor chip surface (Fig. 1A and B; Supplementary Figs S4–S6). Rho-PE signal fully covered the region corresponding to the sensor chip flow cells and localized in the YZ plane as a continuous layer with constant thickness (~20 μm) (Fig. 1C). In contrast, the detected CF fluorescence signal intensity was close to background noise and did not co-localize specifically with Rho-PE, suggesting that the probe was released during SUV deposition. The residual CF signal was localized underneath the Rho-PE signal layer, as depicted in the Z-stack image analysis.

To further characterize the lipid surface formed on the L1 sensor chip, we performed a FRAP experiment on the sensor chip surface. FRAP corresponds to the time-resolved analysis of fluorescence signal recovery in an intentionally bleached area^21^. Free diffusing fluorophores will stochastically diffuse into the bleached area and induce a time-dependent signal recovery. If the fluorophores are spatially confined, inside deposited lipid vesicles, for instance, the signal will not recover as a function of time. Rho-PE fluorescence signal was bleached up to 80% of its initial value, in different lipid compositions (Fig. 1D–H). Rho-PE signal recovery after bleaching was slow in POPC and POPC:Chol:SM (1:1:1) membranes and negligible in POPC:Chol (2:1) and POPC:DPPC (1:1) membranes.

Combined, the 2D confocal imaging and FRAP results suggest that lipid vesicles fully cover the flow cell regions of the L1 sensor chip surface, losing their integrity upon deposition. However, lipid diffusion appears to remain spatially confined, possibly due to the formation of discontinuous lipid membrane patches or incomplete fusion.

### Validation of the steady-state model

**Figure 2:**
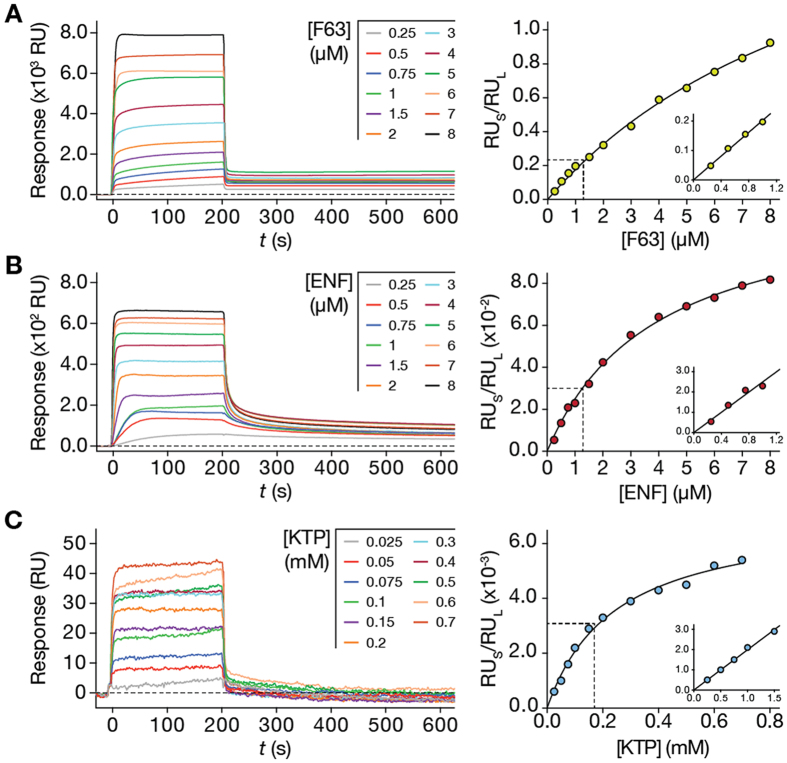
Application of the steady-state model to F63 (**A**), ENF (**B**) and KTP (**C**) SPR lipid membrane (POPC SUV) interaction data. Left: SPR sensorgrams having association time intervals of 200 s and dissociation time intervals of 800 s (truncated to emphasize the association phase and dissociation decay). Right: Data fitting with the partition formalisms (equation (S22) and (S23)). RU_S_ values were collected from individual F63, ENF and KTP sensorgrams at 200 s of the association phase for each solute concentration. RUs values were computed relative to the RU_L_ values at each concentration. The dashed line highlights regions close to linearity, as depicted in each inset. Equation (S22) was fitted to the full concentration range while equation (S23) was fitted to the data in each inset. The presented results represent one of three independent replicates.

The steady-state model is a mathematical approach to rigorously quantify solute membrane partition using current SPR lipid bilayer-solute association response data. The partition formalism of equation (S22), can be directly fitted to RU_S_/RU_L_ vs [S]_W_ data sets to retrieve *K*_p_ and σ values. While σ values are very important for specific applications (membrane-targeting antibiotics, for instance^25^), *K*_p_ is a fundamental membrane interaction parameter studied for both endogenous and therapeutic molecules. To validate the applicability of our model to different molecules with variable properties, we studied a protein, F63, a peptide, ENF, and a low molecular weight molecule, KTP, of known *K*_p_. The respective *K*_p_ values towards POPC lipid membranes have been previously determined using a fluorescence spectroscopy approach^14^,^26^,^27^. SPR membrane interaction experiments were performed for F63, ENF and KTP, using deposited POPC SUV. The flow and association time interval were optimized so that steady-state response plateaus were achieved in each case. This was to allow solute partition to reach an equilibrium. Response steady-states were reached after 200 s in association phase for F63, ENF and KTP with a 5 μL/min flow speed (Fig. 2A,B and C, left panel). Using the association response values at 200 s and the total lipid deposition response, we plotted RU_S_/RU_L_ as a function of the injected sample concentration (Fig. 2A,B and C, right panels). Equation (S22) was fitted to the experimental data. The respective *K*_p_ and σ values are represented in Table 1. The *K*_p_ values obtained for F63, ENF and KTP, 11.04 × 10^3^, 6.41 × 10^3^ and 0.10 × 10^3^, respectively, are in agreement to those in the literature: 3.27 × 10^3^, 3.20 × 10^3^ and 0.66 × 10^3^, respectively. Variations within the same order of magnitude are expected due to the differences in sensitivity intrinsic to the spectroscopic signals that are used. The obtained σ parameter values were approximately 9, 50 and 69 for F63, ENF and KTP, respectively. The value obtained for F63 is within the range expected for membrane-interacting peptides that tend to self-associate. KTP and ENF have σ typical of charged membrane-active peptides^25^.

At low solute concentrations, each of the RU_S_/RU_L_ vs [S]_W_ partition plots is close to linearity (Fig. 2, right panel insets). This behaviour is characteristic of solute partition far from membrane saturation conditions (i.e. equation (S3)). If steric restrictions are absent, equation (S23) may be directly fitted to the data. The *K*_p_ determined through this approach were, 11.6 × 10^3^, 5.29 × 10^3^ and 0.06 × 10^3^ for F63, ENF and KTP, respectively. These values are in close agreement with the data in Table 1, which accounts for saturation in the full solute concentration range. This linear model is a simplification suitable for specific cases of low membrane saturation.

### Steady-state and Dissociation model application

**Figure 3:**
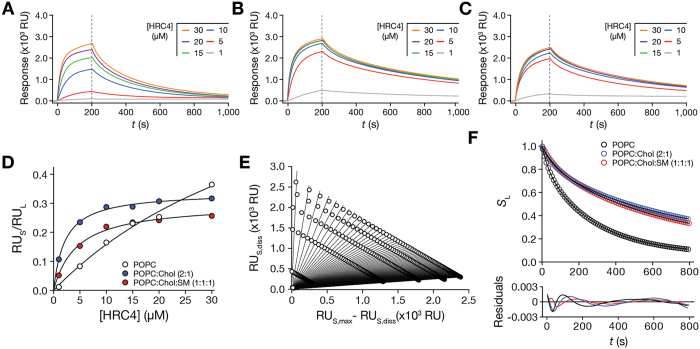
HRC4 membrane interactions studied using SPR and analysing the data using the steady-state and dissociation models. SPR sensorgrams of HRC4 interacting with deposited POPC **(A)**, POPC:Chol (2:1) **(B)** and POPC:Chol:SM (1:1:1) **(C)** SUV. The association time was 200 s and the dissociation time was 800 s in each sensorgram. Association and dissociation phases are separated by a vertical dashed line. **(D)** HRC4 partition extent towards POPC, POPC:Chol (2:1) and POPC:Chol:SM (1:1:1) SUV. RU_S_ values were collected from individual HRC4 sensorgrams at 200 s and divided by RU_L_ at each concentration. The curves correspond to one representative replicate of the fit of equation (S22) to the experimental data. **(E)** Sensorgram dissociation data treatment with equation (S31). Each fitted curve (equation (S31)) contains data relative to an individual time point from the dissociation phase. The presented data is relative to the experiments with POPC membranes. **(F)** HRC4 fractional dissociation from POPC, POPC:Chol (2:1) and POPC:Chol:SM (1:1:1) SUV. Membrane associated HRC4 fractions, *S*_L_, were plotted as a function of the dissociation time. The curves correspond to the best fit of equation (1) to one of three independent replicates. The respective residuals plots are represented. Experiments were performed in triplicate.

Application of the steady-state and dissociation models will have most impact in the study of newly developed molecules, such as pharmaceuticals, with unknown *K*_p_ and σ values. To test the integrated model approach, we studied HRC4, a recently developed antiviral peptide that inhibits MV infection at the entry stage^17^. HRC4 was designed to interact with lipid membranes, through its conjugated Chol domain^28^. Still, its membrane partition properties remain unknown. In order to fully describe the interaction of the peptide with membranes of different properties, the steady-state and dissociation models were applied to partition studies using SPR with POPC, POPC:Chol (2:1), and POPC:Chol:SM (1:1:1) SUV (Fig. 3A,B and C). These lipid compositions are representative of cell membranes, viral envelope and liquid ordered (*l*_o_) raft-like domains, respectively.

The experimental conditions (flow and duration of association time interval) were optimized so that sensorgram response plateaus were observed during the association phase. This can be achieved through adjusted contact times and flow speed. Association response values were collected at 200 s, divided by the total SUV deposition response and fitted with equation (S22) (Fig. 3D). The retrieved partition parameters are summarized in Table 2. *K*_p_ values were 2.30 × 10^3^ for POPC membranes, 15.35 × 10^3^ for POPC:Chol (2:1) membranes and 6.66 × 10^3^ for POPC:Chol:SM (1:1:1) membranes. σ were approximately 16, 39 and 45 for POPC, POPC:Chol (2:1) and POPC:Chol:SM (1:1:1) membranes, respectively.

Dissociation was followed for 800 s (200 to 1000 s after initial sample injection) to adequately monitor solute release from the membrane. Equation (S31) was fitted to sensorgram data sets obtained at different time points (Fig. 3E; Supplementary Fig. S7) and the *S*_L_ values were plotted as a function of time (Fig. 3F). Equation (S28) did not adequately fit to the experimental data (Supplementary Fig. S8). Because of the tendency of Chol to segregate in Chol-rich domains^29^,^30^, we hypothesized that an aggregated HRC4 population may be contributing to the complex dissociation behaviour from lipid membranes. This would explain why equation (S28) failed to adequately adjust to the dissociation profiles. Considering that the dissociation response is the contribution of two separate peptide populations, a monomeric and an aggregated, equation (S28) must be adapted:


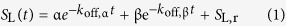


and:


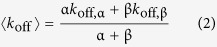


α and β are the fractional contributions of each population to the measured response of each solute population; *k*_off,α_ and *k*_off,β_ are the dissociation constants of each solute population; <*k*_off_> is the weight-averaged *k*_off_. Equation (1) adequately fit the data, as indicated by the respective residual plot (Fig. 3F). The fitting parameters are summarized in Table 2. Because we lack specific information on the respective behaviour of each HRC4 population, we cannot attribute α and β to either the monomeric or aggregated fractions. Our results show that HRC4 has increased partition and slower dissociation from Chol-containing membranes. It is known that the cholesterol moieties tend to associate to cholesterol in membranes, which justifies the increased partition towards cholesterol-containing membranes. Cholesterol organizes in *l*_o_ domains, which explains why dissociation is slower when compared to POPC. In agreement, dissociation is also slow when rigid raft-like membranes are used. Likewise, the fraction of retained (*S*_L,r_) peptide drugs is equally increased in ordered cholesterol-rich and raft-like membranes.

## Discussion

We have developed a SPR analytical methodology to assess solute partition towards lipid membranes. Our approach is based on two complementary mathematical models, derived from classical partition formalisms that can be directly applied to SPR sensorgram data in order to retrieve quantitative interaction parameters. One model, called the steady-state model here, was designed for association phase response data analysis at the partition equilibrium and allows for the experimental determination of *K*_p_ and σ. The other model, called the dissociation model, is intended for kinetic evaluation of dissociation phase response data and allows quantification of *k*_off_ and *S*_L,r_. Combined, these models achieve broad range analysis of SPR sensorgram data, both in equilibrium and non-equilibrium states.

The models imply initial assumptions based on the fundamentals of SPR experimental setups: (i) Response is proportional to the mass of solute interacting with lipid bilayers. Previous observations of protein interaction with dextran matrixes^31^, suggested that sample detection follows a linear relationship within the detection range of SPR equipments. (ii) The partition equilibrium is only achieved at the association response steady-state. During these periods, the sensorgram signal remains constant due to the balanced distribution of solute between the aqueous and lipid phase. (iii) Upon reaching the partition equilibrium, the solute concentrations will remain constant in each phase.

In order to prepare a stable and reproducible lipid phase for our experiments, we followed a widely accepted protocol for SUV deposition on a Biacore L1 sensor chip surface^32^,^33^, in which vesicles are captured through entropic interactions with an acylated dextran matrix. However, there has been controversy regarding the organization of the lipid surface formed through this approach^23^,^24^. Through confocal fluorescence microscopy imaging and FRAP experiments we observed that fluorescently-labelled vesicles fully cover the sensor chip flow cell regions and lose their content upon deposition, independently of the studied lipid composition (Fig. 1, Supplementary Figs S4–S6). Interestingly, a surface composed of discontinuous lipid membranes may be formed as opposed to a homogeneous supported bilayer. This hypothesis is supported by FRAP studies performed with intact lipid vesicles^34^,^35^,^36^. It is possible that SUVs partially retain their morphology albeit releasing their content to the external medium, as previously observed in experiments under shear stress^37^ or with glass surfaces^38^. This would explain why probe fluorescence signal recovery in FRAP experiments exhibited extremely slow kinetics, in contrast with typical results in continuous supported lipid bilayers^39^. There is also no sign of loose vesicle diffusion in solution, which would lead to a fast recovery of the bleached area^40^. These results are in line with prior studies with the L1 sensor chip^23^ and support the assumption that A_L_ ≈ A_total_, and consequently, *m*_S_ ≈ *m*_S, L_ is also valid. Accordingly, we have assumed that unspecific solute binding to the dextran matrix is residual and thus negligible.

The steady-state model also assumes that response signals are equally proportional to the respective lipid and solute mass (*k*_S_ ≈ *k*_L_). The SPR detection principle is based on local changes in the medium refractive index near the sensor chip gold surface and consequent shifts in the incident wave reflection angles^41^. As a result, solute detection is directly dependent on its respective refractive index increment (d*n*/d*c*), which modulates the relative sensitivity of the technique for each chemical species. Proteins and liposomes display similar d*n*/d*c* values, respectively, an average of 0.185 and 0.16, and should, in principle, elicit a similar response within the SPR detection range^42^. However, one cannot exclude that uncharacterized molecules such as small organic compounds may display a significantly different d*n*/d*c* parameter.

We validated applicability of the steady-state model using three distinct molecules with known *K*_p_ values (Fig. 2). F63, ENF and KTP were chosen to cover a wide range of molecular weights (Supplementary Table S1). Using this model, we were able to determine the *K*_p_ of F63, ENF and KTP towards POPC membranes (Table 1). Small discrepancies between retrieved SPR-based *K*_p_ and published values obtained from independent techniques may be explained by the different membrane model systems used in both cases. While the value found in the literature was determined through fluorescence spectroscopy in the presence of 100 nm-diameter large unilamellar vesicles (LUV), SPR experiments were performed with fused SUV. Additionally, fluorescence methodologies are dependent on the responsiveness of fluorescent amino acid residues, such as tryptophan, which may contribute for small differences between techniques.

If only *K*_p_ is desired, a simplified version of the steady-state model (equation (S23)) can be applied to the linear region in each partition plot (Fig. 2, right panel insets). We applied the steady-state model in full to determine σ for each molecule in addition to *K*_p_. F63 showed a lower σ compared to ENF and KTP, which suggests a higher solute:lipid ratio in saturating conditions probably indicative of membrane-induced self-association. The σ of ENF and KTP were, respectively, 50 and 69. These values are comparable to data previously obtained for the antimicrobial peptide omiganan, which has a σ of 66, and other membrane-active peptides^25^.

It should be stressed that σ is an extremely difficult parameter to calculate using conventional techniques and methodologies. Membrane saturation demands high [S]_W_. When conventional spectroscopic techniques are used, the spectroscopic signal results from an ensemble-average of the signal originating from aqueous solute, S_W_, and lipid-inserted solute, S_L_. When [S]_W_ is high in conditions in which 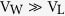, the signal originated from S_W_ is much higher than the signal originated from S_L_, which is in practice totally masked and therefore undetectable. This makes σ impossible to obtain using conventional techniques. Taking fluorescence emission or circular dichroism (CD) spectroscopies as examples^13^: saturation demands high [S]_W_ and experimental setups determine 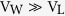 so 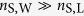. In practice, the measured fluorescence intensity emission or CD absorbance is due entirely to the aqueous solute. S_L_ has nearly nil contribution to the spectroscopic signal response reading, which makes σ very difficult to be obtained. Calculation of σ with fluorescence spectroscopy methodologies requires, besides a fluorescent solute sensitive to polarity, very high *K*_p_ and very high σ so that saturation is achieved at relatively low [S]_W_. Even so, the methodology is time-consuming and automation requires specific adaptations^43^. The proposed methodology in this paper is straightforward and overcomes the limitations of conventional techniques.

The partition models were used to analyse the interaction of HRC4 with POPC, POPC:Chol (2:1) and POPC:Chol:SM (1:1:1) SUV (Fig. 3). HRC4 is a novel anti-MV peptide having a Chol moiety but not yet studied for its membrane-level activity. Association and dissociation sensorgram data were analysed using the integrative approach of combined steady-state and dissociation models. HRC4 has increased affinity towards membrane compositions containing Chol, as shown by *K*_p_ values (Table 2). This preference correlates with the ability of Chol to condensate fluid lipid bilayers and assemble into *l*_o_ Chol-rich domains, such as lipid rafts.

HRC4 dissociation does not follow first order kinetics. σ suggests the derivatized peptide may self-associate in membranes, which may be the basis of complex dissociation kinetics. Additionally, HRC4 is able to self-assemble in solution^44^. We modified equation (S24) to account for two distinct peptide populations, either monomeric or aggregated, for instance, and adequately fitted the resulting equation (1) to the dissociation data (Fig. 3F). HRC4 dissociates faster from POPC membranes and is more retained in membranes containing Chol. This is shown by both the <*k*_off_> and *S*_L,r_ parameters (Table 2). The conjugated Chol moiety promotes HRC4 retention and binding to membranes that contain Chol. These observations may be biologically relevant since viral envelopes are cholesterol-rich and the peptide selectively targets viral envelopes. Moreover, when designing custom lipid-based drug delivery systems, it is important to consider the maximal drug load in the drug delivery system (σ), the total amount needed to achieve maximal drug load (dictated by *K*_p_), the rate of release of the drug in plasma <*k*_off_> and the retained fraction in the carrier (*S*_L,r_).

Although the proposed method provides a powerful tool that expands the realm of SPR applications and addresses specific needs that cannot be satisfied by other techniques, it has certain limitations. Deposited vesicle surface properties may vary depending on the lipid composition used, stressing the importance of preliminary confocal microscopy studies. The models are not applicable to solutes with very slow association kinetics that fail to reach an association steady-state under experimental conditions. Additionally, for specific membrane-translocating molecules with low *K*_p_ such as cell-penetrating peptides (CPP), accumulation of solutes beneath the deposited vesicle membranes may interfere with the assessment. Finally, as with other techniques, detergent-like molecules that can damage lipid membrane integrity, cannot be studied through this approach.

The goal of the present study was to provide an alternative approach to quantify molecular partition towards lipid membranes using a well-defined set of physical chemistry parameters. We expect our work to contribute to make SPR as versatile and useful in unravelling the fundamental parameters of solute-lipid membrane interaction as it is now to obtain binding constants in ligand-receptor stoichiometric interactions.

## Additional Information

**How to cite this article**: Figueira, T. N. *et al*. Quantitative analysis of molecular partition towards lipid membranes using surface plasmon resonance. *Sci. Rep.* **7**, 45647; doi: 10.1038/srep45647 (2017).

**Publisher's note:** Springer Nature remains neutral with regard to jurisdictional claims in published maps and institutional affiliations.

## Supplementary Material

Supplementary Information

## Figures and Tables

**Table 1:** Partition parameters of F63, ENF and KTP interaction with POPC membranes: comparison of results obtained with the steady-state model and published data.

Molecule	Literature		Steady-state Model (±SD)	
	*K*_p_ × 10^3^ (±SD)	Reference	*K*_p_ × 10^3^	σ
F63	3.27 (±0.70)	14	11.04 (±0.28)	9.3 (±1.2)
ENF	3.20 (±0.20)	26	6.41 (±0.50)	50.4 (±2.0)
KTP	0.66 (±0.23)	27	0.10 (±0.01)	69.0 (±6.3)

**Table 2:** Steady-state and dissociation model parameters obtained from the analysis of HRC4 interaction with POPC, POPC:Chol (2:1) and POPC:Chol:SM (1:1:1) membranes.

Lipid Mixture	Steady-state Model (±s.d.)		Dissociation Model (±s.d.)					
	*K*_p_ × 10^3^	σ	*k*_off,α_ × 10^−3^ (*s*^−1^)	α	*k*_off,β_ × 10^−3^ (*s*^−1^)	β	<*k*_off_> × 10^−3^ (*s*^−1^)	*S*_L,r_
POPC	2.54 (±0.29)	25.9 (±8.8)	3.8 (±0.3)	0.74 (±0.01)	26.0 (±2.0)	0.19 (±0.01)	8.30 (±0.8)	0.07 (±0.01)
POPC:Chol (2:1)	11.4 (±3.41)	41.4 (±2.7)	1.6 (±0.1)	0.64 (±0.05)	16.2 (±0.1)	0.16 (±0.01)	5.56 (±0.5)	0.20 (±0.05)
POPC:Chol:SM (1:1:1)	7.24 (±0.74)	48.7 (±3.6)	2.0 (±0.3)	0.70 (±0.02)	17.1 (±2.3)	0.16 (±0.02)	4.78 (±0.9)	0.14 (±0.01)
