# Supplementary material for: Quantitative analysis of molecular partition towards lipid membranes using surface plasmon resonance
Source: Sci Rep. 2017 Mar 30;7:45647. doi: 10.1038/srep45647 (PMC5372468; doi:10.1038/srep45647)
Supplement: Supplementary Information [file srep45647-s1.doc]

**Supplementary Information**

Quantitative analysis of molecular partition towards lipid membranes using surface plasmon resonance

Tiago N. Figueira^1^, João M. Freire^2^, Catarina Cunha-Santos^3^, Montserrat Heras^4^, João Gonçalves^3^, Anne Moscona^5^, Matteo Porotto^5^, Ana Salomé Veiga^1^, Miguel A.R.B. Castanho^1^*

*^1^Instituto de Medicina Molecular, Faculdade de Medicina, Universidade de Lisboa, Portugal*

*^2^Institut Pasteur, Unité de Virologie Structurale, Département de Virologie, F-75724 Paris Cedex 15, France*

*^3^Instituto de Investigação do Medicamento (iMed.ULisboa), Faculdade de Farmácia, Universidade de Lisboa, Portugal*

*^4^Laboratori d’Innovació en Processos i Productes de Síntesi Orgànica (LIPPSO), Departament de Química, Universitat de Girona, Spain*

*^5^Department of Pediatrics, Columbia University Medical Center, New York, USA*

1. **Molecules description**

**Supplementary Table S1** – Chemical composition and molecular weight of the molecules studied in the present work

| **Molecule** | **Chemical Composition*** | **Molecular Weight (Da)** |
| --- | --- | --- |
| F63 | NH_2_-EQLVLTQQPASVEAAPGGTVTIKCSGGGSDSTDYAKGYANYSGG  GSWYQQKPGHPPKLLIYKASTLASGVSSRFSGSGSGTEFTLTISDLKSA  DAATYYCSGGGSSTYAYAYSSGAYSGGGSGGGTELEIKSGQAGQSGG  GGSHHHHHHHHGAYPYDVPDYAS-OH | 16440 |
| ENF | Ac-YTSLIHSLIEESQNQQEKNEQELLELDKWASLWNWF-NH_2_ | 4492 |
| KTP | NH_2_-YR-OH | 337 |
| HRC4 | ([Ac-PPISLERLDVGTNLGNAIAKLEDAKELLESSDQILRGSGSGC-Maleimide-PEG_4_]-NH_2_)_2_-Chol | 10159 |

*Amino acids are represented in single letter code. (Ac – acetylated N-terminus; PEG – polyethylene glycol)

1. **Data analysis model development**

Two mathematical models have been developed to assess and quantify molecular partition towards lipid membranes in a SPR experimental setting. They allow *K*_p_ and *k*_off_ determination through complementary steady-state and kinetic analysis, respectively. σ, the lipid:solute molar ratio at membrane saturation is also determined, as well as the retained solute fraction, *S*_L,r_. The mathematical methodology will be described in detail in the present section. When mentioned, sensorgrams are regarded in full, i.e. kinetic profiles composed of an association and dissociation phase, measured in response units (RU)^1^.

*Steady-state model – K*_p_ *and* σ *determination*

The partition equilibrium is commonly described as the distribution of a given solute between two separate and immiscible phases^2^. When solute partition occurs between an aqueous and a lipid phase, the equilibrium may be described as follows:

| $\text{[S]}_{\text{W}}$⇄$\text{[S]}_{\text{L}}$ | (S1) |
| --- | --- |

where [S]_W_ and [S]_L_ correspond to the solute concentration in the aqueous and lipid phase, respectively. This equilibrium can be generalized to most biological lipid membrane association processes that result from solute adsorption or insertion into lipid bilayers. The partition constant (*K*_p_) is the parameter that quantifies the relative solute distribution in each phase:

| $K_{\text{p}}=\frac{\frac{n_{\text{S,L}}}{\text{V}_{\text{L}}}}{\frac{n_{\text{S,W}}}{\text{V}_{\text{W}}}}=\frac{\text{[S]}_{\text{L}}}{\text{[S]}_{\text{W}}}$ | (S2) |
| --- | --- |

where *n*_S,X_ are the moles of solute in aqueous (X = W) or lipid phase (X = L) and V_W_ and V_L_ are, respectively, the volumes of the aqueous and lipid phase. This formalism was first introduced by Nernst^3^.

In flow-based SPR systems, samples at a given concentration [S]_W_ are continuously injected over a pre-formed lipid surface. When the partition equilibrium is reached and solute concentrations remain constant in both phases, the concentration of membrane-bound solute, [S]_L_, corresponds to:

| $\text{[S]}_{\text{L}}=K_{\text{p}}\text{[S]}_{\text{W}}$ | (S3) |
| --- | --- |

Because of the continuous injection flow, [S]_W_ is constant for a given assay and independent of solute uptake by the membrane. For different assays in which the [S]_W_ is varied, equation (S3) can be modified to consider the concentrations of the solute in the initial (i) and final (f) assays in both phases:

| $\text{([S]}_{\text{L,f}}-\text{[S]}_{\text{L,i}}\text{)}=K_{\text{p}}\text{([S]}_{\text{W,f}}-\text{[S]}_{\text{W,i}}\text{)}$ | (S4) |
| --- | --- |

and thus,

| $\text{Δ[S]}_{\text{L}}=K_{\text{p}}\text{Δ[S]}_{\text{W}}$ | (S5) |
| --- | --- |

Linear partition relationships such as equation (S5) are only valid in the absence of phase saturation^4^. Saturation occurs from the steric restrictions imposed by gradual solute partition towards a limited phase volume. So, inducing a solute concentration variation in aqueous phase, Δ[S]_W_, may not necessarily lead to a linear increase in the solute concentration of the lipid phase, Δ[S]_L_.

Due to the limited lipid vesicles surface, lipid phase saturation is expected in SPR experiments, at high solute concentrations. To account for this phenomenon, we have introduced a saturation correcting factor (*f*_sat_) in equation (S5):

| $\text{Δ[S]}_{\text{L}}=K_{\text{p}}\text{Δ[S]}_{\text{W}}f_{\text{sat}}$ | (S6) |
| --- | --- |

defined as:

| $f_{\text{sat}}=\frac{\text{V}_{\text{L,F}}}{\text{V}_{\text{L}}}=\frac{\text{V}_{\text{L}}-\text{V}_{\text{L,O}}}{\text{V}_{\text{L}}}$ | (S7) |
| --- | --- |

V_L,F_ corresponds to the free lipid phase volume while V_L,O_ corresponds to the lipid phase volume occupied by the solute. This factor corrects for the negative deviation to the linear partition regime, imposed by lipid phase occupation and consequent steric restrictions. Consequently, the partition equilibrium will follow a linear relationship as long as $\text{V}_{\text{L,F}}\approx\text{V}_{\text{L}}$. Alternatively, if $\text{V}_{\text{L,F}}<<\text{V}_{\text{L}}$, partition will be negatively affected.

In a saturating lipid membrane, V_L,O_ is^4^:

| $\text{V}_{\text{L,O}}=\text{σγ}_{\text{L}}n_{\text{S,L}}$ | (S8) |
| --- | --- |

where, γ_L_ is the lipid molar volume and σ is the lipid to solute ratio ($n_{\text{L}}/n_{\text{S,L}}$) at the saturation point. Combining equations (S6), (S7), and (S8):

| $\text{Δ[S]}_{\text{L}}=K_{\text{p}}\text{Δ[S]}_{\text{W}}\frac{\text{V}_{\text{L}}-\text{σγ}_{\text{L}}n_{\text{S,L}}}{\text{V}_{\text{L}}}$ | (S9) |
| --- | --- |

Considering that $n_{\text{S,L}}/\text{V}_{\text{L}}$ is [S]_L_:

| $\frac{\text{Δ[S]}_{\text{L}}}{\text{Δ[S]}_{\text{W}}}=K_{\text{p}}\text{(1}-\text{σγ}_{\text{L}}\text{[S]}_{\text{L}}\text{)}$ | (S10) |
| --- | --- |

In the context of continuous functions, infinitely small concentration variations may be considered and equation (S10) integrated:

| $\text{[S]}_{\text{L}}=\frac{K_{\text{p}}\text{[S]}_{\text{W}}}{1+\text{σγ}_{\text{L}}K_{\text{p}}\text{[S]}_{\text{W}}}$ | (S11) |
| --- | --- |

While equation (S3) holds true for linear partition systems, in which solutes are in dilute concentrations in each phase, equation (S11) accounts for the steric restrictions involved in solute lipid phase saturation. The $\frac{1}{1+\text{σγ}_{\text{L}}K_{\text{p}}\text{[S]}_{\text{W}}}$ multi-parameter component, which we have designated as steric restriction factor, determines how much the partition equilibrium will deviate from linearity. This will depend on the solute concentration in the aqueous phase, solute affinity for a specific lipid phase and the limiting saturation conditions. If $\text{σγ}_{\text{L}}K_{\text{p}}\text{[S]}_{\text{W}}<<1$, equation (S11) resembles equation (S3) and saturation has little to nil influence in solute partition. Conversely, if $\text{σγ}_{\text{L}}K_{\text{p}}\text{[S]}_{\text{W}}>>1$, [S]_L_ reaches values close to the saturation concentration limit, [S]_L,sat_, and therefore the system is governed by saturation. In SPR experiments, due to the limited lipid phase volume, it is common to reach near-saturation conditions.

Even though SPR does not allow the direct measurement of bound solute concentrations, specifically [S]_L_, the sensorgram response values are correlated to the amount of interacting solute^5^:

| $\text{[S]}_{\text{L}}\propto\text{RU}_{\text{S}}$ | (S12) |
| --- | --- |

where RU_S_ corresponds to the solute membrane association response units. The partition equilibrium (equation (S11)) is only valid after sensorgrams reach a response plateau (i.e. $\text{ΔRU}_{\text{S}}\approx0$). Given the nature of SPR detection and experimental principles^6^, RU can be re-written as the detected solute/analyte mass (*m*) per area unit (A):

| $\text{RU}=k\frac{m}{\text{A}}$ | (S13) |
| --- | --- |

in which *k* is a proportionality constant. Under these considerations, the response of a specific solute partitioning towards deposited lipid membranes can be postulated as:

| $\text{RU}_{\text{S}}=k_{\text{S}}\frac{m_{\text{S}}}{\text{A}_{\text{total}}}\approx k_{\text{S}}\frac{m_{\text{S,L}}}{\text{A}_{\text{L}}}$ | (S14) |
| --- | --- |

where *m*_S_ is the solute mass, *m*_S,L_ is the detected solute mass partitioned to the lipid phase, A_total_ is the total sensor chip surface area, A_L_ is the deposited lipid phase surface area, and *k*_S_ is a proportionality constant. Equation (S14) is valid depending on two relative assumptions: $\text{A}_{\text{L}}\approx\text{A}_{\text{total}}$, the deposited lipid phase surface area is equivalent to the total sensor chip area, and $m_{\text{S}}\approx m_{\text{S,L}}$, unspecific interactions between the solute and the sensor chip surface are negligible, under experimental conditions. Experimental confirmation of the validity of the first and second assumptions will be further commented in the results and discussion sections.

Considering that $m_{\text{S.L}}=n_{\text{S,L}}\text{M}_{\text{S}}$, where M_S_ is the molecular mass of the solute, and $\text{A}_{\text{L}}=\text{V}_{\text{L}}/h_{\text{L}}$, where *h*_L_ is the lipid bilayer cross section length, equation (S14) becomes:

| $\text{RU}_{\text{S}}=k_{\text{S}}\frac{n_{\text{S,L}}\text{M}{}_{\text{S}}h_{\text{L}}}{\text{V}_{\text{L}}}$ | (S15) |
| --- | --- |

Because $n_{\text{S,L}}/\text{V}_{\text{L}}$ is [S]_L_:

| $\text{RU}_{\text{S}}=k_{\text{S}}\text{M}{}_{\text{S}}h_{\text{L}}\text{[S]}_{\text{L}}$ | (S16) |
| --- | --- |

To further simplify equation (S16), we consider the molar volume of the lipid, γ_L_, is given by:

| $\text{γ}_{\text{L}}=\frac{\text{V}_{\text{L}}}{n_{\text{L}}}$ | ((S17) |
| --- | --- |

Where *n*_L_ corresponds to the moles of lipid. Replacing $\text{V}_{\text{L}}=\text{A}_{\text{L}}h_{\text{L}}$ and $n_{\text{L}}=m_{\text{L}}/\text{M}_{\text{L}}$ in equation (S17):

| $h_{\text{L}}=\frac{\text{γ}_{\text{L}}m_{\text{L}}}{\text{M}_{\text{L}}\text{A}_{\text{L}}}$ | (S18) |
| --- | --- |

Following the assumption that $\text{A}_{\text{L}}\approx\text{A}_{\text{total}}$, i.e. the lipid phase surface area is approximately equal to the total deposition area, equation (S14) can be adapted to the lipid response values, RU_L_:

| $h_{\text{L}}=\frac{\text{γ}_{\text{L}}\text{RU}_{\text{L}}}{\text{M}_{\text{L}}k_{\text{L}}}$ | (S19) |
| --- | --- |

Equation (S19) can then be used in equation (S16), which is transformed into:

| $\text{RU}_{\text{S}}=\frac{k_{\text{S}}}{k_{\text{L}}}\text{RU}{}_{\text{L}}\text{γ}_{\text{L}}\frac{\text{M}_{\text{S}}}{\text{M}_{\text{L}}}\text{[S]}_{\text{L}}$ | (S20) |
| --- | --- |

RU_L_ is the total lipid deposition response, M_L_ is the molecular mass of the lipids and *k*_L_ is a proportionality constant. For the sake of simplicity, we have considered that $k_{\text{S}}\approx k_{\text{L}}$. Through this approximation we assume that lipids (in the form of SUVs) and solutes modulate the refractive properties of the solvent in a similar fashion. Both liposomes and proteins, for instance, exhibit similar refractive index increment ($\text{d}n/\text{d}c$) values (0.16 and an average 0.185, respectively^7^), a parameter descriptive of these properties. Accordingly, they should induce comparable response variations within the SPR detection range. The equivalent may be considered for uncharacterized small molecules or polymers, notwithstanding slight deviations in the model’s predictions. Thus, equation (S20) can be simplified and rearranged into:

| $\text{[S]}_{\text{L}}=\frac{\text{RU}_{\text{S}}}{\text{RU}_{\text{L}}}\frac{\text{M}_{\text{L}}}{\text{M}_{\text{S}}\text{γ}_{\text{L}}}$ | (S21) |
| --- | --- |

Combining equations (S11) and (S21), we reach a SPR partition analysis formalism:

| $\frac{\text{RU}_{\text{S}}}{\text{RU}_{\text{L}}}=\frac{\text{γ}_{\text{L}}K_{\text{p}}\frac{\text{M}_{\text{S}}}{\text{M}_{\text{L}}}\text{[S]}_{\text{W}}}{1+\text{σγ}_{\text{L}}K_{\text{p}}\text{[S]}_{\text{W}}}$ | (S22) |
| --- | --- |

Equation (S22) can be directly applied to lipid deposition and solute association response data and represents a straightforward approach to determine *K*_p_ and σ values using SPR. Most parameters are known constants and *K*_p_ and σ are fitting parameters. Respectively, these provide quantitative data on solute affinity towards lipid membranes and on the respective saturating conditions.

Under specific conditions of low membrane saturation, i.e. very low solute concentrations, we may assume that steric restrictions at the membrane level are negligible. Concurrently, this translates into $\text{σγ}_{\text{L}}K_{\text{p}}\text{[S]}_{\text{W}}\approx0$, which implies a linear partition relationship, simplified from equation (S22) as:

| $\frac{\text{RU}_{\text{S}}}{\text{RU}_{\text{L}}}=\text{γ}_{\text{L}}K_{\text{p}}\frac{\text{M}_{\text{S}}}{\text{M}_{\text{L}}}\text{[S]}_{\text{W}}$ | (S23) |
| --- | --- |

This formalism can be applied to linear datasets, in which equation (S22) proves ambiguous. However, information relative to the σ parameter is lost in this case.

*Dissociation model – k*_off_ *and S*_L,r_ *determination*

In the previous section, we defined a partition formalism to be applied to SPR sensorgram response data at the association steady-state phase. During the SPR dissociation phase, the solvent (running buffer) replaces the solute solution and solute molecules dissociate from the lipid phase, at specific rate. Quantitatively assessing the unbinding kinetics of solutes should provide insight into the releasing rate and retention of partitioned solutes.

Progressive solute dissociation from lipid membranes can be described as a partition equilibrium between an aqueous and a lipid phase, in which concomitantly the aqueous solute concentration is depleted due to solvent flow:

| $\text{S}_{\text{L}}$⇌$\text{S}_{\text{W}}$→$\text{S}_{\text{eluted}}$ | (S24) |
| --- | --- |

S_eluted_ represents the solute eluted by solvent flow.

The rate of [S]_L_ variation is:

| $\frac{\text{d[S]}_{\text{L}}}{\text{d}t}=-k_{\text{off}}\text{[S]}_{\text{L}}+k_{\text{on}}\text{[S]}_{\text{W}}$ | (S25) |
| --- | --- |

*k*_off_ and *k*_on_ are the rates of membrane release and insertion, respectively. Being *k*_e_, the rate of the elution process, much higher than *k*_on_ such that $\text{[S]}_{\text{W}}\approx0$:

| $\frac{\text{d[S]}_{\text{L}}}{\text{d}t}\approx-k_{\text{off}}\text{[S]}_{\text{L}}$ | (S26) |
| --- | --- |

Dissociation follows a first order rate law dependent on [S]_L_. The corresponding integrated rate law is:

| $\text{[S]}_{\text{L}}\text{(}t\text{)}=\text{([S]}_{\text{L,i}}-\text{[S]}_{\text{L,r}}\text{)e}^{-k_{\text{off}}t}+\text{[S]}_{\text{L,r}}$ | (S27) |
| --- | --- |

Solute dissociation progresses from an initial state, [S]_L,i_, corresponding to the maximum solute concentration within the lipid phase, and evolves through time to a constant final retained solute concentration, [S]_L,r_. If dissociation is total, $\text{[S]}_{\text{L,r}}=0$. Membrane-associated solute fraction, *S*_L_, calculated through normalization of [S]_L_(*t*) values to [S]_L,i_, $S_{\text{L}}=\text{[S]}_{\text{L}}\text{(}t\text{)}/\text{[S]}_{\text{L,i}}$, can also be used to monitor dissociation from the lipid phase:

| $S_{\text{L}}\text{(}t\text{)}=\text{(1}-S_{\text{L,r}}\text{)}e^{-k_{\text{off}}t}+S_{\text{L,r}}$ | (S28) |
| --- | --- |

where, *S*_L,r_ is the retained solute fraction at $t=\infty$, $S_{\text{L,r}}=\text{[S]}_{\text{L,r}}\text{(}t=\infty\text{)}/\text{[S]}_{\text{L,i}}$. *S*_L_(*t*) values are more easily correlated with SPR experimental data than the respective [S]_L_(*t*). Because *S*_L_(*t*) is a dimensionless and fractional parameter, it can be directly calculated from sensorgram dissociation response values (RU_S,diss_(*t*)), normalized to the maximum association response (RU_S,max_, which corresponds to the initial dissociation state) :

| $S_{\text{L}}\text{(}t\text{)}=\frac{\text{RU}_{\text{S,diss}}\text{(}t\text{)}}{\text{RU}_{\text{S,max}}}$ | (S29) |
| --- | --- |

Because sensorgram dissociation response values are not only time-dependent but also sample concentration-dependent, a generalized version of the model can be developed:

| $S_{\text{L}}\text{(t)}=\frac{\text{RU}_{\text{S,diss}}\text{(}t\text{,[S]}_{\text{W}}\text{)}}{\text{RU}_{\text{S,max}}\text{([S]}_{\text{W}}\text{)}}$ | (S30) |
| --- | --- |

At a given dissociation time point $t=\text{τ}$, *S*_L_(*τ*) values can be obtained through data fitting with the following linearized formalism:

| $\text{RU}_{\text{S,diss}}\text{(}\tau\text{,[S]}_{\text{W}}\text{)}=\frac{S_{\text{L}}\text{(}\tau\text{)}}{1-S_{\text{L}}\text{(}\tau\text{)}}\text{(RU}_{\text{S,max}}\text{([S]}_{\text{W}}\text{)}-\text{RU}_{\text{S,diss}}\text{(}\tau\text{,[S]}_{\text{W}}\text{))}$ | (S31) |
| --- | --- |

Thus, *S*_L_(*τ*) is obtained for a series of injected concentrations, [S]_W_, by fitting equation (S31) to the RU_S,diss_(*τ,*[S]_W_) vs (RU_S,max_([S]_W_) – RU_S,diss_(*τ,*[S]_W_)) data. Finally, the *S*_L_(*t*) vs *t* dissociation curve data points are fitted with equation (S28), which allows the experimental determination of the *k*_off_ and *S*_L,r_ parameters.

Application of the steady-state and dissociation models as an integrated approach enables quantitative analysis of SPR solute-membrane interaction data. Still, the independent application of each model to separate data sets is also possible. Equations (S22) and (S28) can be directly fitted to experimental values and require minimal data processing. The resulting *K*_p_, σ and *k*_off_ parameters are descriptive of solute partition quantification and represent a more rigorous approach compared to current methods based on *K*_d_.

1. **Small Unilamellar Vesicle (SUV) Characterization**

**
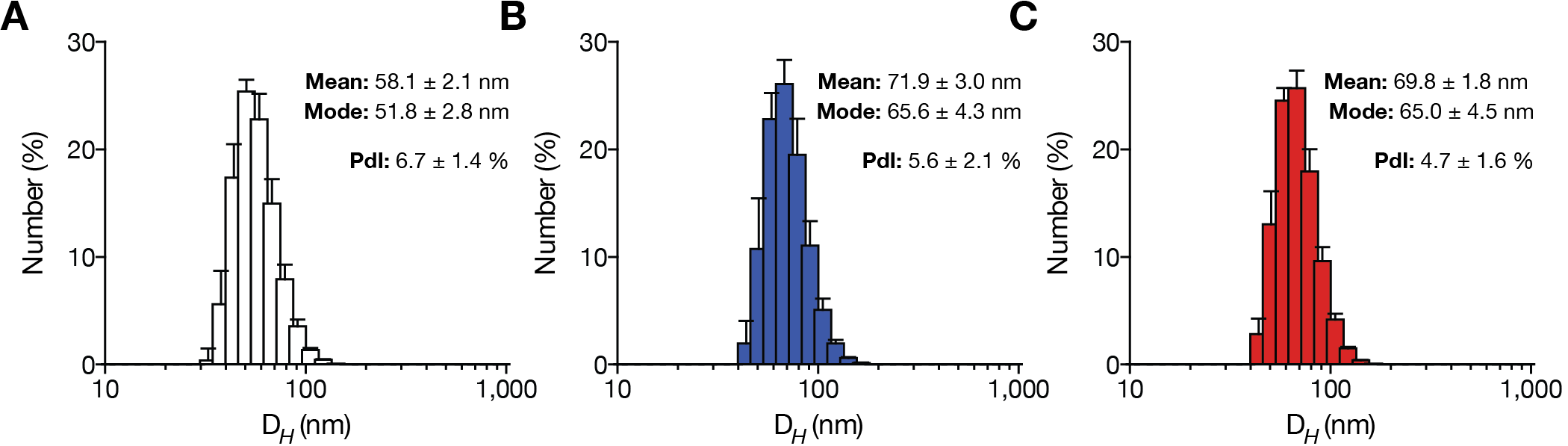
**

**Supplementary Fig. S1 – SUV particle size characterization.** Hydrodynamic diameter (D*_H_*) profiles of POPC (A), POPC:Chol (2:1) (B) and POPC:Chol:SM (1:1:1) (C) SUVs (200 μM), obtained from dynamic light scattering (DLS) analysis. The D*_H_* mean, D*_H_* mode and polydispersity index (PdI) are shown for each SUV composition. Measurements were performed in a Malvern Zetasizer Nano ZS (Worcestershire, UK) and consisted of 15 individual technical replicates, each corresponding to an averaged autocorrelation curve. Samples were incubated at 25 ºC for a period of 5 min before measurements started. Error bars correspond to the standard deviation.

**
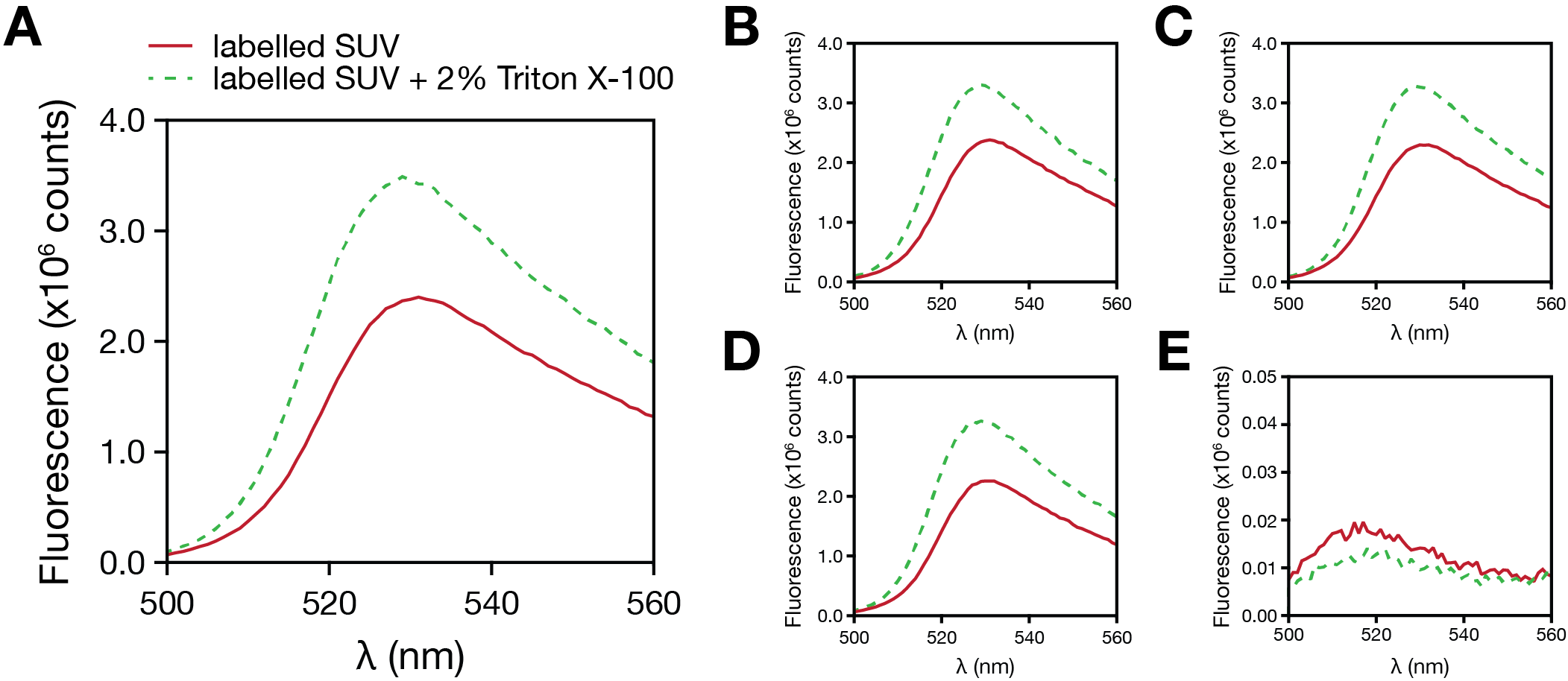
**

**Supplementary Fig. S2 – Confirmation of CF encapsulation in POPC (A), POPC:Chol (2:1) (B), POPC:Chol:SM (1:1:1) (C), POPC:DPPC (1:1) (D) SUV.** Fluorescence emission spectra of a sample of free and SUV encapsulated CF (500 μM) fluorophore populations. SUV with different lipid compositions were prepared as described in the methods section and contained both Rho-PE and CF probes. Spectra were acquired in an Edinburgh FLS920 spectrofluorometer using 0.5 cm optical path quartz cells. The excitation wavelength was fixed at 480 nm. Excitation and emission slits were 2 and 5 nm, respectively. Experiments were performed at 25 ºC. CF release from the SUV lumen was induced by addition of Triton X-100 up to 2% (v/v). The resulting increase in CF spectra fluorescence intensity is attributed to probe dequenching, following SUV disruption. Spectra were corrected for the respective dilution. **(E)** The fluorescence emission spectra of a POPC SUV sample containing Rho-PE but not CF was used as a control. Only residual fluorescence was detected in this case.

1. **Confocal Microscopy**

**
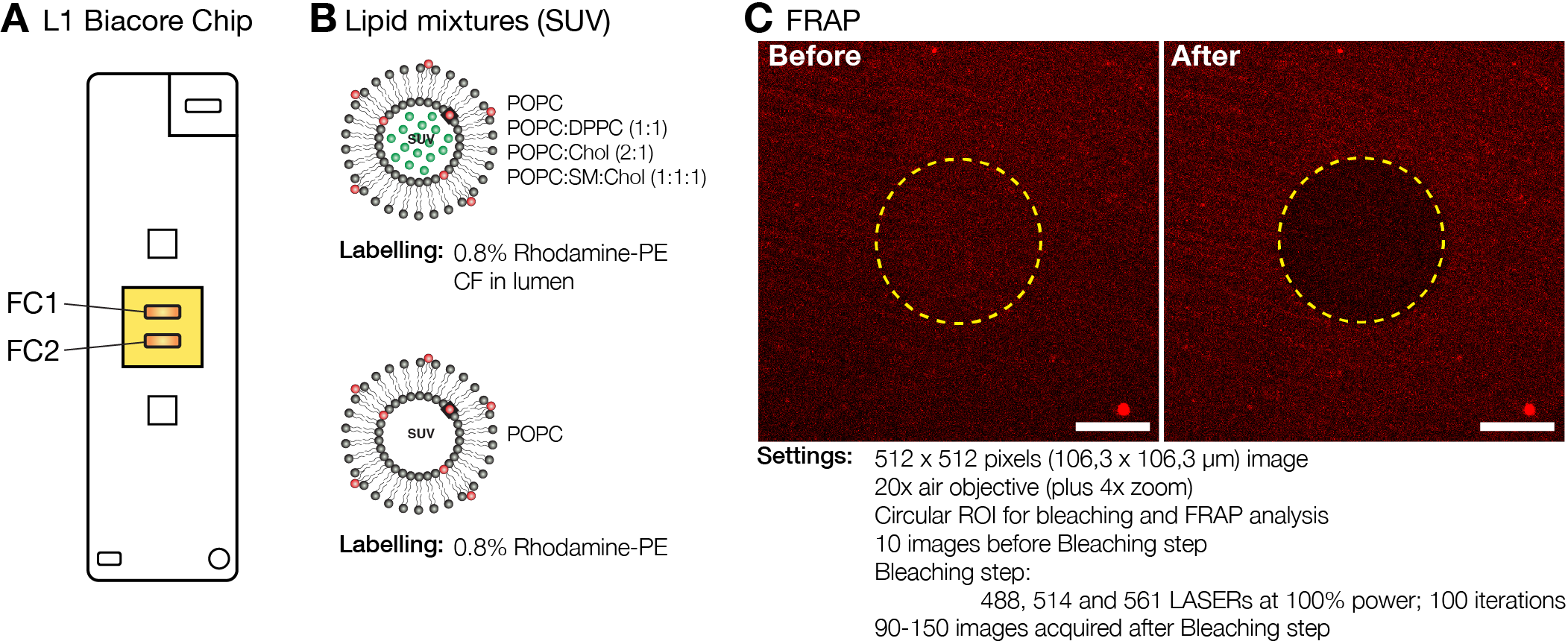
**

**Supplementary Fig. S3 – Lipid desposition studies on a L1 sensor chip. (A)** schematic representation of Flow Cells (FC) 1 and 2 of the L1 sensor chip. **(B)** Identification of the lipid mixtures and molar proportions used in the studies and FRAP experiments. **(C)** 4x Zoom images using a 20x magnification objective highlighting the Rho-PE membrane labelling and fluorescence before and after bleaching protocol for FRAP studies. The experiments were performed using the following protocol: 10 frames were collected before bleaching of the 20 µm radius circular ROI (yellow) with 100% of 488, 514 and 561 laser intensity for 100 iterations (approx. 40 s). After bleaching 90-150 images were acquired according to the lipid mixture studied and due to fluorescence recovery/sample burning percentage.


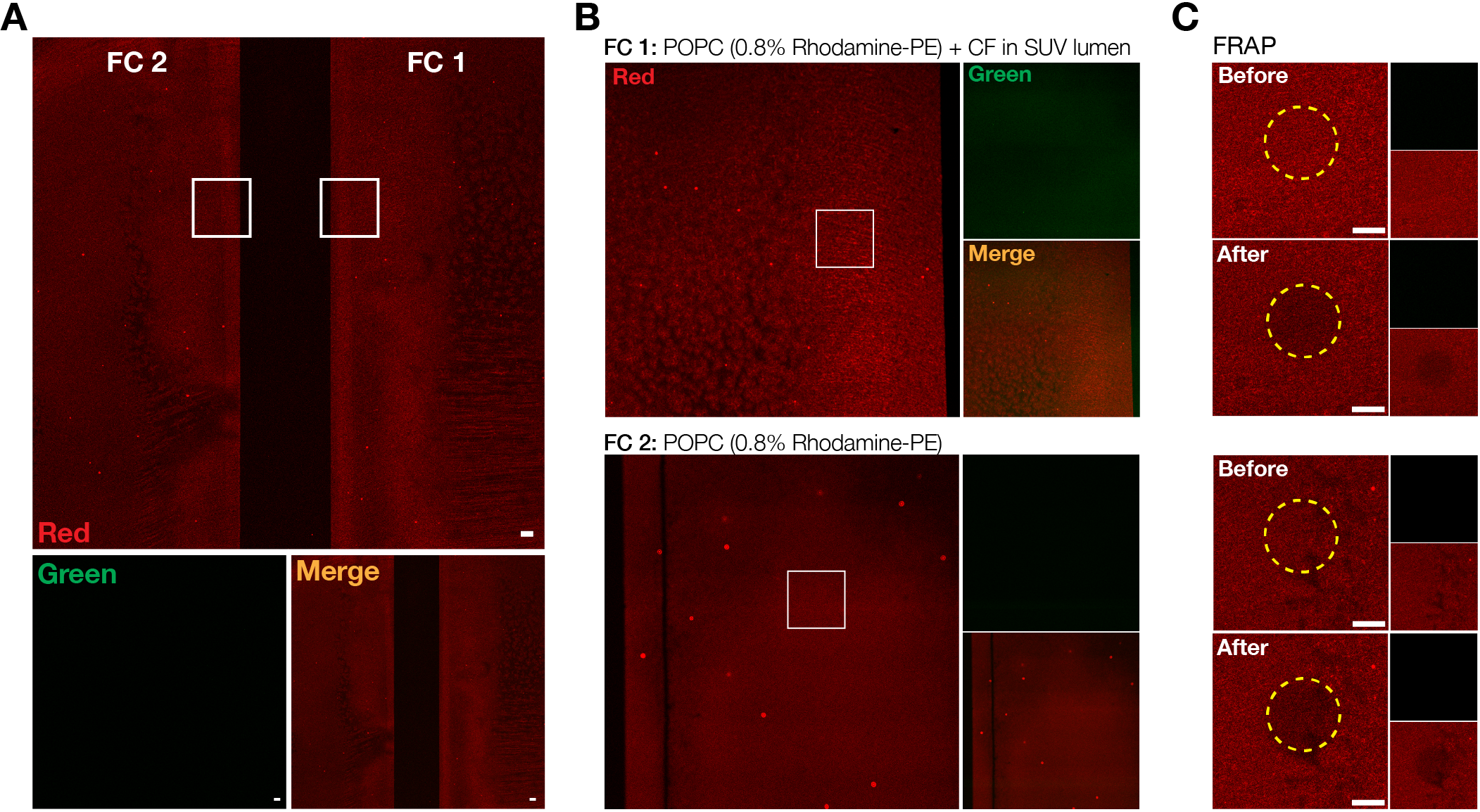


**Supplementary Fig. S4 – Observation of the lipid surface formed on a L1 sensor chip. (A)** Confocal microscopy image of Flow Cells (FC) 1 and 2 of the L1 sensor chip using a 10x magnification objective. FC 1 was covered with POPC SUV labelled in the membrane with 0.8% Rho-PE (red panel) and CF (green panel) at the SUV lumen. FC 2 was covered with POPC only labelled in the bilayer with 0.8% Rho-PE. The merge between the Rho-PE and CF signals is also depicted. **(B)** Individual 20x magnification confocal images of FC 1 (top panel) and FC 2 (bottom panel) of the L1 sensor chip surface (at the ROI highlighted in **A**). Red, green and merge channels are shown. **(C)** 4x Zoom images at the ROI highlighted in **B** of both FC 1 and FC 2 before and after applying the Bleaching protocol to perform FRAP studies. 10 frames were collected before bleaching of the 20 µm radius circular ROI (yellow) with 100% of 488, 514 and 561 laser intensity for 100 iterations (approx. 40 s).


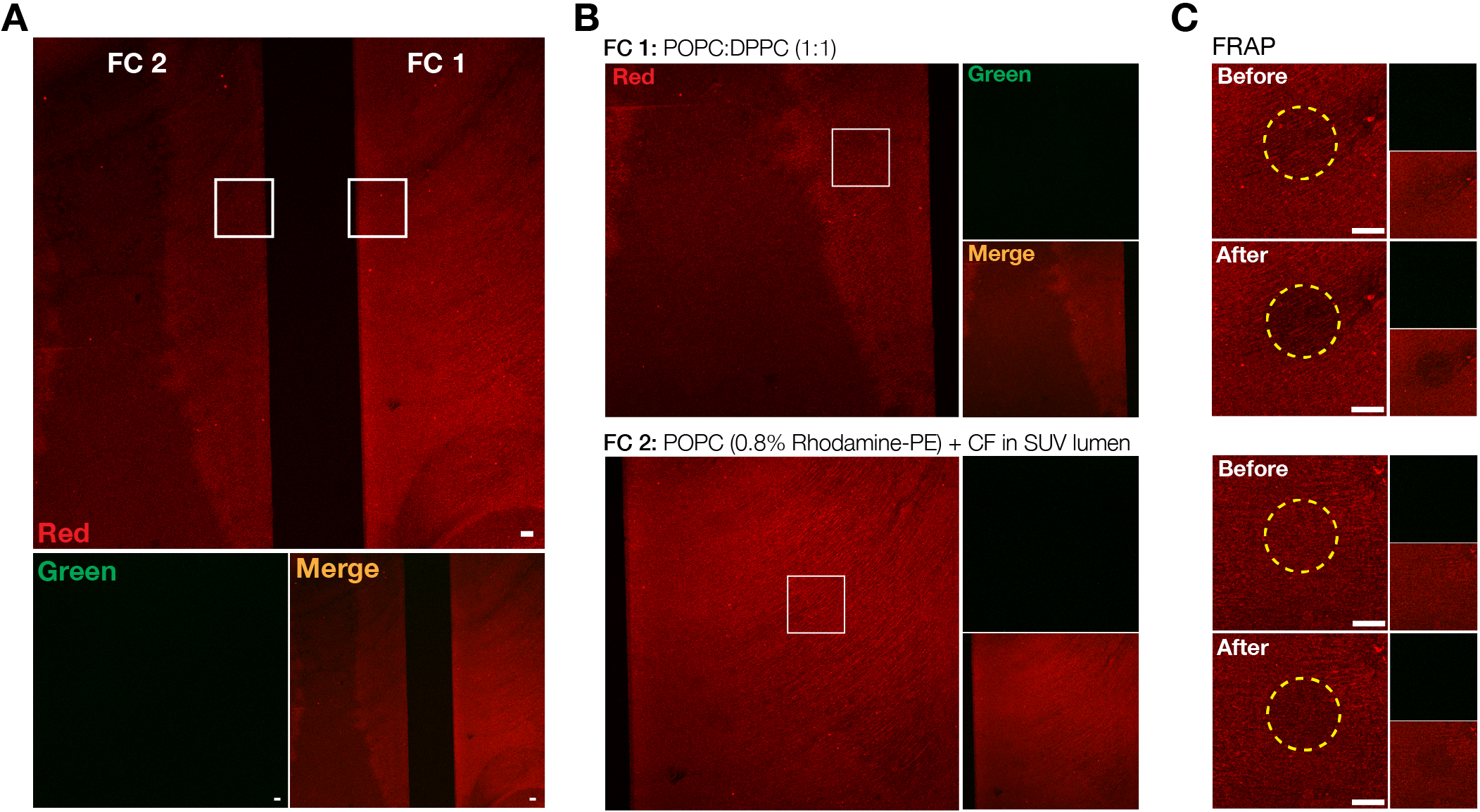


**Supplementary Fig. S5 – Observation of the lipid surface formed on a L1 sensor chip. (A)** Confocal microscopy image of Flow Cells (FC) 1 and 2 of the L1 sensor chip using a 10x magnification objective. FC 1 was covered with POPC:DPPC (2:1) SUV and FC 2 with POPC. SUV membranes and lumen were fluorescently labelled with Rho-PE (red panel) and CF (green panel) fluorescent probes, respectively. The merge between the Rho-PE and CF signals is also depicted. **(B)** Individual 20x magnification confocal images of FC 1 (top panel) and FC 2 (bottom panel) of the L1 sensor chip surface (at the ROI highlighted in **A**). Red, green and merge channels are shown. **(C)** 4x Zoom images at the ROI highlighted in **B** of both FC 1 and FC 2 before and after applying the Bleaching protocol to perform FRAP studies. 10 frames were collected before bleaching of the 20 µm radius circular ROI (yellow) with 100% of 488, 514 and 561 LASER intensity for 100 iterations (approx. 40 s).


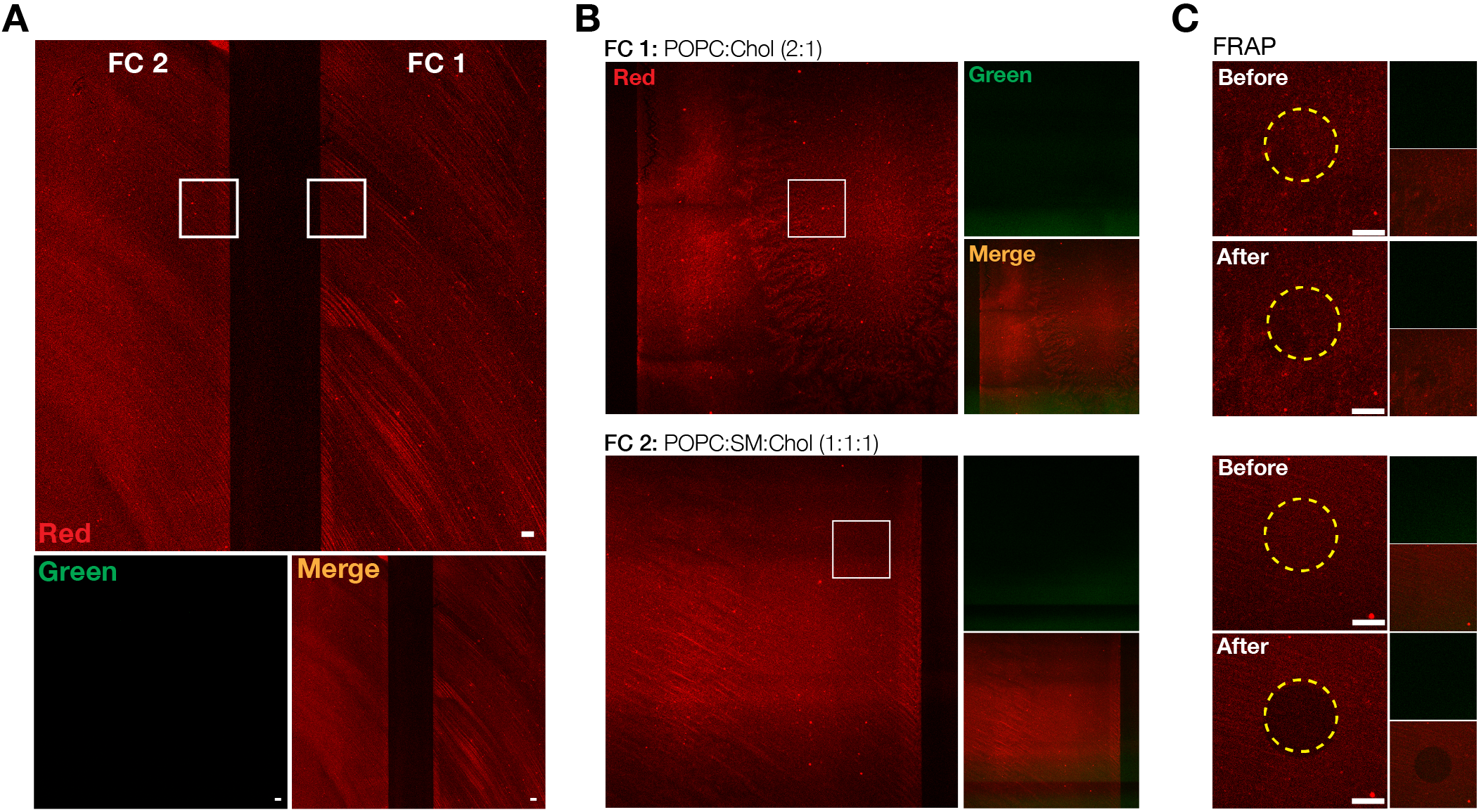


**Supplementary Fig. S6 – Observation of the lipid surface formed on a L1 sensor chip. (A)** Confocal microscopy image of Flow Cells (FC) 1 and 2 of the L1 sensor chip using a 10x magnification objective. FC 1 was covered with POPC:Chol (2:1) SUV and FC 2 with POPC:SM:Chol (1:1:1). SUV membranes and lumen were fluorescently labelled with Rho-PE (red panel) and CF (green panel) fluorescent probes, respectively. The merge between the Rho-PE and CF signals is also depicted. **(B)** Individual 20x magnification confocal images of FC 1 (top panel) and FC 2 (bottom panel) of the L1 sensor chip surface (at the ROI highlighted in **A**). Red, green and merge channels are shown. **(C)** 4x Zoom images at the ROI highlighted in **B** of both FC 1 and FC 2 before and after applying the Bleaching protocol to perform FRAP studies. 10 frames were collected before bleaching of the 20 µm radius circular ROI (yellow) with 100% of 488, 514 and 561 laser intensity for 100 iterations (approx. 40 s).

1. **Model Data Fitting**


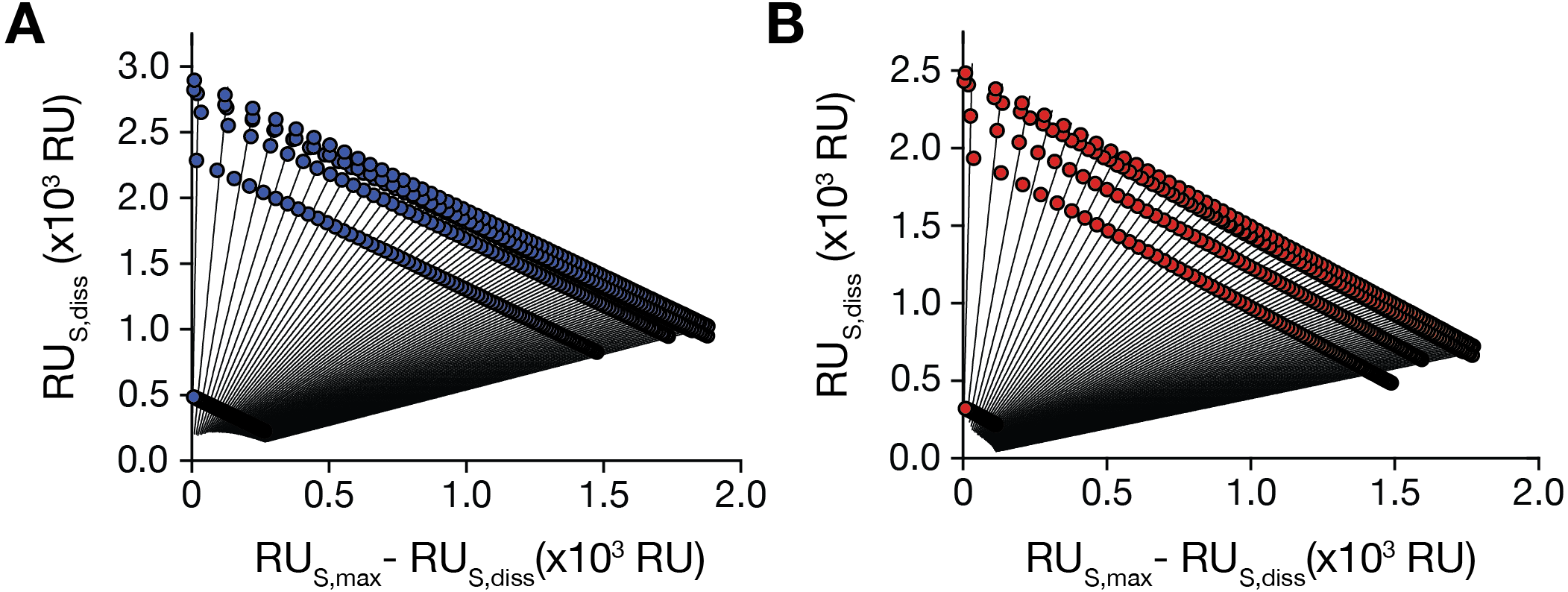


**Supplementary Fig. S7 – HRC4 membrane dissociation data treatment with equation (S31).** SPR sensorgram data of HRC4 dissociation from POPC:Chol (2:1) **(A)** and POPC:Chol:SM (1:1:1) **(B)** membranes plotted as RU_S,diss_ vs (RU_S,max_ – RU_S,diss_) data sets. Equation (S31) was fitted to individual data sets obtained at distinct dissociation time points (between 0 and 800 s of the dissociation phase). These were composed of 6 points, corresponding to each HRC4 concentration studied. The obtained *S*_L_ values (fitting parameter of equation (S31)) were used to quantitatively study HRC4 dissociation from membranes.


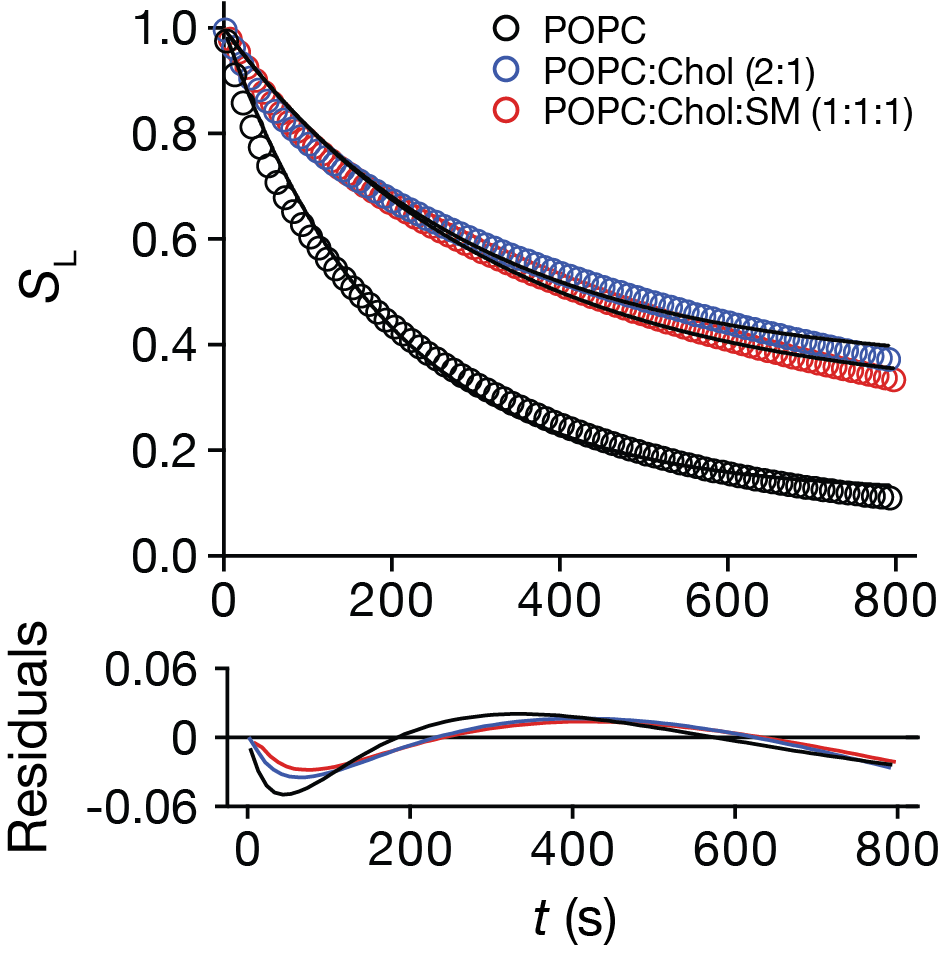


**Supplementary Fig. S8 – Fitting equation (S28) to HRC4 fractional membrane dissociation data.** HRC4 membrane associated fractions, *S*_L_, were determined from POPC, POPC:Chol (2:1) and POPC:Chol:SM (1:1:1) membrane dissociation sensorgram data and plotted as a function of the dissociation time. Equation (S28) was fitted to the data through a non-linear regression analysis. A significant deviation from each data set is observed in both the regressions (top) and residuals plots (bottom).

**References**

1. Cooper, M. A. Optical biosensors in drug discovery. *Nat Rev Drug Disc* **1,** 515–528 (2002).

2. Santos, N. C., Prieto, M. & Castanho, M. A. R. B. Quantifying molecular partition into model systems of biomembranes: an emphasis on optical spectroscopic methods. *BBA* **1612,** 123–135 (2003).

3. Nernst, W. Verteilung eines Stoffes zwischen zwei Lösungsmitteln und zwischen Lösungsmittel und Dampfraum. *Z. phys. Chem.* **8,** 110–139 (1891).

4. Melo, M. N. & Castanho, M. A. R. B. Omiganan interaction with bacterial membranes and cell wall models. Assigning a biological role to saturation. *BBA* **1768,** 1277–1290 (2007).

5. Stenberg, E., Persson, B. & Roos, H. Quantitative determination of surface concentration of protein with surface plasmon resonance using radiolabeled proteins. *J Colloid Interf Sci* **143,** 513–526 (1991).

6. Daghestani, H. N. & Day, B. W. Theory and applications of surface plasmon resonance, resonant mirror, resonant waveguide grating, and dual polarization interferometry biosensors. *Sensors* **10,** 9630–9646 (2010).

7. Theisen, A., Deacon, M. P., Johann, C. & Harding, S. E. *Refractive Increment Data-Book for Polymer and Biomolecular Scientists*. (Nottingham University Press, 2000).
